# Improving the Security and Confidentiality in the Internet of Medical Things Based on Edge Computing Using Clustering

**DOI:** 10.1155/2021/6509982

**Published:** 2021-10-26

**Authors:** Anita Hatamian, Mohammad Bagher Tavakoli, Masoud Moradkhani

**Affiliations:** ^1^Department of Electrical Engineering, Arak Branch, Islamic Azad University, Arak, Iran; ^2^Department of Electrical Engineering, Ilam Branch, Islamic Azad University, Ilam, Iran

## Abstract

Families, physicians, and hospital environments use remote patient monitoring (RPM) technologies to remotely monitor a patient's vital signs, reduce visit time, reduce hospital costs, and improve the quality of care. The Internet of Medical Things (IoMT) is provided by applications that provide remote access to patient's physiological data. The Internet of Medical Things (IoMT) tools basically have a user interface, biosensor, and Internet connectivity. Accordingly, it is possible to record, transfer, store, and process medical data in a short time by integrating IoMT with the data communication infrastructure in edge computing. (Edge computing is a distributed computing paradigm that brings computation and data storage closer to the sources of data. This is expected to improve response times and save bandwidth. A common misconception is that edge and IoT are synonymous.) But, this approach faces problems with security and intrusion into users' medical data that are confidential. Accordingly, this study presents a secure solution in order to be used in the IoT infrastructure in edge computing. In the proposed method, first the clustering process is performed effectively using information about the characteristics and interests of users. Then, the people in each cluster evaluated by using edge computing and people with higher scores are considered as influential people in their cluster, and since users with high user interaction can publish information on a large scale, it can be concluded that, by increasing user interaction, information can be disseminated on a larger scale without any intrusion and thus in a safe way in the network. In the proposed method, the average of user interactions and user scores are used as a criterion for identifying influential people in each cluster. If there is a desired number of people who are considered to start disseminating information, it is possible to select people in each cluster with a higher degree of influence to start disseminating information. According to the research results, the accuracy has increased by 0.2 and more information is published in the proposed method than the previous methods.

## 1. Introduction

The Internet of Things is a new concept in the world of information and communication technology; in fact, it is a modern technology that provides the ability to send and receive data through communication networks, whether the Internet or intranet for any entity [1]. Smart devices are all part of an emerging category called the Internet of Things, or IoT for short. At a basic level, the Internet of Things deals with connecting different objects through the Internet and communicating with each other to achieve its goal of providing a more efficient and intelligent experience.

In the meantime, improving health is considered to be the ultimate goal of economic, social, and technical progress, and rapid population growth and aging will change the world significantly. These factors have put a lot of pressure on food suppliers and health systems around the world, and the advancement of new IoT technology is expected to provide appropriate and potential solutions to these challenges. Therefore, the use of IoT for health can certainly be effective in strategic research roadmap in medicine and the use of IoT. Nowadays, the advent of various wearable devices has enabled people to monitor their health anywhere and anytime through the Internet of Medical Things (IoMT) [2]. Accordingly, IoT-based wearable devices will become a key part of human health in the not-too-distant future. IoMT  not only is able to monitor physiological information about the human body through wearable devices but also can send the results of this information to a remote monitoring center or family doctor, or even generate emergency alerts. It should be noted that IoMT can help physicians to suggest treatment programs through recommender systems.

This method can significantly reduce the time of medical examination, improve diagnostic efficiency, and save human resources [3]. But now, it is impractical to transfer information from wearable devices (where they were created) to the cloud infrastructure (where they are processed) because the cloud-centric processing method results in high communication delays and reduces data transfer rates between IoT devices and IoT devices with potential users.

In order to accomplish the previously stated goal, the concept of edge computing has recently been proposed, which makes it possible to process IoT services to process data near data sources and data sinks instead of engaging in the cloud [4]. This solution reduces communication delays and makes better use of the computing, storage, and network resources that are currently available. It can also reduce runtime and power consumption, which can be very useful for using the Internet of Things in health and medicine.

Given that edge computing is a new research topic, robust integrated solutions to its various challenges, including security, and secure routing, have not yet been proposed. However, one of the problems with using such systems is that unauthorized people infiltrate the system and use its information or perform unauthorized manipulations. This feature allows unauthorized access to existing data and performing manipulations in these networks [5]. Therefore, this study examines the process of detecting intrusion into the Internet of Medical Things using clustering methods in the edge computing infrastructure and the results will be compared with some other methods in this field.

The most important contributions of this study include the following:

  Providing a clustering-based routing method for use in the Internet of Medical Things (IoMT).
  Using edge computing to reduce delays in sending important medical information to emergency centers safely
  An interactive and structural approach is used to increase the accuracy within the clusters in order to increase efficiency and increase security. In this way, the influential nodes can be identified with higher accuracy.

## 2. Research Background

The study in [6] reviewed security and confidentiality in the IoMT. Accordingly, firstly security and privacy problems in various parts of the IoMT have been investigated. The existing solutions in the field of security and privacy problems, which provide the suitable context for solving these challenges for the IoT infrastructure, were reviewed. According to the results of this study, there is a need for new methods in the process of providing security and intrusion detection in IoMT.

The challenges and methods of detecting malware intrusion in the Internet of Things were reviewed in [7]. This study aimed to conduct a comprehensive review of existing problems and solutions. The presence of various botnets in IoMT infrastructures has led to attacks on the security of an infrastructure, including comprehensiveness, confidentiality, and availability. These can reduce the spread of new infrastructure such as IoMT. Accordingly, firstly, the types of malware attacks and how they work have been examined.

Then, an IoT-based architecture with various security protocols is examined for use in deploying and securing the IoMT infrastructure. The study in [8] proposed a secure framework using the Internet of Medical Things that also improves energy consumption. This method is designed to reduce communication overhead and energy consumption between medical sensors that process and transmit health information.

On the other hand, the proposed method is able to secure the medical data of patients against unwanted access. According to the evaluation results, energy consumption is reduced by 29%, and productivity is increased by 18%. A fog computing-based (Fog computing or fog networking, also known as fogging, is an architecture that uses edge devices to carry out a substantial amount of computation, storage, and communication locally and routed over the Internet backbone.) attack detection system using MOA-WMA algorithm is presented in [9].

The proposed model consists of several parts. In preprocessing, the input data is first processed and converted into a format that can be used in machine learning, and the required features are extracted from them and then sent to the feature selection section, where the required features are selected for accurate detection of intrusion and enter the next section after passing a filter to remove additional information, for modeling in this phase using a multilayer neural network. Finally, the next steps are done based on this modeling and learning that is done with the help of training data. The evaluation results indicate an increase in accuracy and efficiency with the help of this method. An in-depth learning-based solution is presented in [10] to extract IoT data features and intrusion detection in IoMT. The deep learning model is combined with intrusion detection technology to help with this approach. This method solves the problem of distributive incompatibility of source and target data. The proposed solution can be used in various neural networks. Accordingly, the problem of data learning can be solved with its help, and abnormal data can be detected more accurately. Thus, evaluations have shown that intrusion into the IoMT can be detected with high accuracy. A Markov model-based approach is presented in [11] to detect anomalies and secure health monitoring systems.

According to the results of this study, due to the continuity of the applied traffic in the network and their interdependence, as a result, abnormal movements in the network can be detected by using the Markov model. This solution is based on data dependence on the network, so it can operate without supervision based on the learning model and be used on wearable sensors with low energy.

Also, unlike other methods that only operate linearly, this method can be performed in nonlinear dimensions as well, which in turn will be able to detect intrusion and anomalies in the network with more power. Finally, the solution is evaluated and it is concluded that, on average, it significantly increases the accuracy of network anomaly detection. A combination of different machine learning methods to detect intrusion into the IoT infrastructure is presented in [12]. Accordingly, six types of machine learning methods (Random Forest, K-Nearest Neighbor Support Vector Machine, Neural Networks, J48, and Decision Table) were used.

This method uses unlabeled data along with monitored methods to increase the accuracy of the IoMT intrusion detection system. This solution teaches a single-layer neural network to categorize samples on unlabeled data. Next, each of the created categories is combined with the initial data set, and retraining is applied to the classification method. According to the results of the research, the accuracy of the solution is high, and also the execution time of the intrusion detection process is reduced.  Table 1 provides a summary of previous methods and the advantages and disadvantages of each method.

## 3. Clustering of the Proposed Method

An interactive approach is used to identify influential nodes in IoT networks, that is, paying attention to interactions between users. User interaction is reflecting a node that receives more tweets but responds less (has fewer retweets) and is a better option to be happy, and in fact, such a node is selected as the influential node. That is, the influential nodes in each association are identified using an analytical relationship based on a combination of structural and interactive approaches. It should be noted that the number of tweets received and responding to each user is used within the database.

### 3.1. Utilizing an Interactive and Structural Approach to Increase Accuracy within Clusters

After identifying the effective users in the previous step, the impact point of each of them is calculated by the following formula:

(1) $$\begin{array}{c} V\left(u_{i},\;u_{j}\right)=\frac{nr_{ij+nr_{ji}}}{NR_{i}+NR_{j}}+\frac{T_{i}}{T_{ij}}, \end{array}$$

where *nr*_*ij*_ is the number of times *u*_*i*_ retweets *u*_*j*_ tweets, *nr*_*ji*_ indicates  the number of times *u*_*j*_ retweets *u*_*i*_ tweets, *NR*_*i*_ is the number of times *u*_*i*_ retweets other users' tweets, *NR*_*j*_  is the number of times *u*_*j*_ retweets the tweets of other users, *T*_*i*_ is the total number of messages received by *i*, and  *T*_*ij*_ is the total number of messages sent from *i* to *j*, each of which is normalized individually.

### 3.2. Research Plan

Given that some of the nodes of a large database are studied (case study) in this study, the research method in this dissertation is a case study.

First, information and theoretical foundations of the research are extracted from valid internal and external sources in the present study. Then the IoT general database Mirai and Bashlite which is available at http://www.archive.ics.uci.edu/mI/datasets/detection_of_iot_botnet_attacks_n_balot is selected for implementation and execution. Data are first categorized by the k-means clustering algorithm; then, intrusion detection and routing are optimized by edge calculations in MATLAB and the results are presented in the form of tables and graphs. Finally, the obtained results are compared with the results of other previous methods.

### 3.3. Problem Solving Method

In the present study, K-means clustering is used to classify the database after selecting the appropriate database. Different forums are identified in the IoT network. In the next step, special attention is paid to the demographic characteristics of users, including their age, gender, and occupation, to identify the forums in the IoT networks. A code between 0 and 1 is assigned to each of these attributes. A kind of clustering algorithm should be used to continue the process of identifying associations based on the mentioned demographic characteristics; the k-means algorithm is selected in the present study.

Then, influential nodes are identified in the IoT networks. Edge computing is used to identify the influential nodes in the IoT networks. The interactive approach in edge computing, that is, interactions between users, has been considered. Interaction between users means a node that receives more information but responds less (has less response) is a better option to be head-cluster, and in fact, such a node is selected as the influential node.

**Figure 1:**
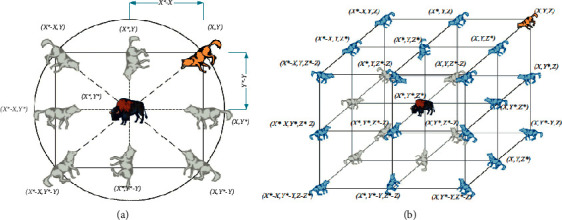
The alpha, beta, and gamma wolf movements.

That is, the influential nodes in each association are identified using an analytical relationship, based on a combination of structural and interactive approaches. It should be noted that the amount of information received and answered for each user is used in the data. By identifying the influential users in the previous step, the impact score of each of them is now calculated and at the end, the obtained values are placed in the objective function which is presented in the next section.  Figure 1 shows the alpha, beta, and gamma wolves movements.

**Figure 2:**
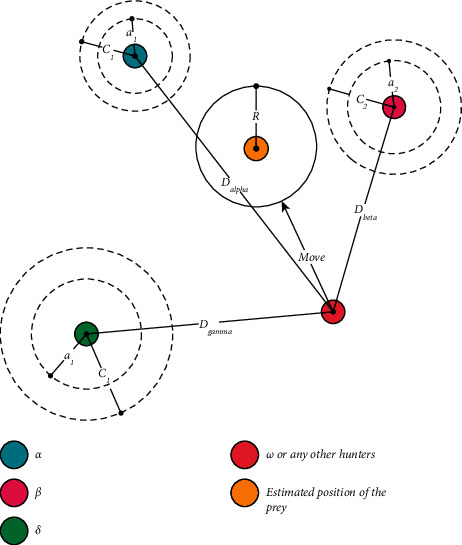
Optimal solutions by alpha, beta, and gamma wolves in the proposed method.

Figure 2 provides the optimal solutions by alpha, beta, and gamma wolves in the proposed method.

**Figure 3:**
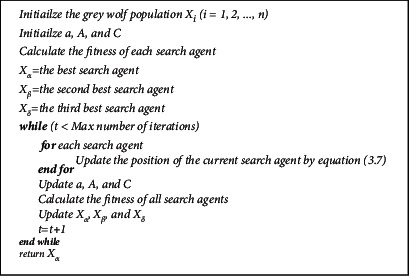
Pseudocode of the gray wolf algorithm.

The pseudocode of the gray wolf algorithm is given in Figure 3.

Table 2 tabulates a part of the study data set.

### 3.4. Objective Function in Edge Computation Algorithm

This study was carried out to increase the accuracy and secure routing of the IoT network using edge computing. Therefore, the objective function is as follows:

(2) $$\begin{array}{c} F\left(u_{i},\;u_{j}\right)=\left(\frac{nr_{ij+nr_{ji}}}{NR_{i}+NR_{j}}\right)*V+1, \end{array}$$

where F is the accuracy in the IoT network and is calculated by edge computing.

In this study, the community includes all users of the database. The sample group includes 1000 users of the database. By definition, the random sampling method is simple [25]. MATLAB (version 2017) was used for data analysis, and results are presented. In this section, simulation results on IoT network data were presented. The simulation consists of two stages.

In the first stage, network forums or clustering of 1000 users is found based on the three characteristics of gender, age, and occupation, and in the next step, the degree of interaction and ranking of clustered users are obtained. The data in this section includes information about these 1000 users and their neighbors, in addition to information about user interactions, shared posts, and user comments about posts. In this method, users are selected with a higher degree of interaction and rank to start the process of disseminating information in each cluster, and information is disseminated through these users to other users.

MATLAB software was used for simulations related to user clustering, and the degree of interaction and user rank were used to perform the relevant simulations.

### 3.5. Platform Used

The platform used Windows 7, 64-bit CPU, 4 GHZ, Core 7 Duo.

### 3.6. Clustering, Number of Clusters, Clustering Effect, Clustering Criteria, and Reason for Using Cummins Algorithm for Clustering

Data clustering is done in this section. Clustering leads to the categorization and ordering of large volumes of information. As a result, it is much easier to study and analyze data in a particular order than to examine large volumes of information without categorization. There are several methods for clustering. The K-means method is one of the data clustering methods in data mining.

This method is a basic method for many other clustering methods (such as fuzzy clustering) although it is a simple method. This method is considered an exclusive method. As a result, this algorithm is used for clustering in this study due to its ease of use. The parameters to be set in the K-means clustering algorithm are given in Table 3.

Figure 1 shows the set parameters, user features, and ignored properties as follows:

  Number of repetitions: 250
  Number of samples: 1000
  Number of clusters: 25
  Distance function: Euclidean distance
  Number of features: 4

**Figure 4:**
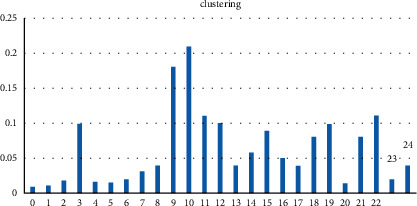
Percentage of users in each cluster.

Information about the number of users in each forum, the percentage of users in each forum, and a comparison between accuracy and number of clusters as well as a comparison between the number of clusters and the time of their next analysis to find effective users are provided in this section. In Figure 4, the columns represent the number of each cluster and the rows the percentage of network users in each cluster. Then, the accuracy of the K-means algorithm was calculated for different numbers. The accuracy of this algorithm increases with the number of different clusters, as the number of clusters increases. The number of 25 clusters is relatively accurate among the number of clusters tested.

Despite the help of user profile information-based clustering in IoT networks, it has the disadvantage of identifying groups with common characteristics because individuals are not interested in disclosing personal information publicly for security reasons.

In this section, the cosine similarity measure was used to perform clustering. (A cosine similarity measure for fuzzy sets [26] is defined as the inner product of two vectors divided by the product of their lengths. This is nothing but the cosine of the angle between the vector representations of the two fuzzy sets.) The cosine similarity measure used by Collins et al. is the similarity measure between two vectors of an interior multiplication space based on the cosine of the angle between them. The cosine of angle of zero degrees is equal to one and is less than one for the other angles. Therefore, it is thus a judgment of orientation and not magnitude. Two vectors with the same direction have a cosine similarity criterion equal to one, two vectors with a 90° angle have a similarity of zero, and two vectors with the opposite direction have a similarity of −1.

The cosine similarity measure is used especially in a positive space with a range of [0, 1]. It should be noted that this range is used for any number of dimensions, typically used in positive dimensions with high dimensions.

First, the cosine distance is defined as

(3) $$\begin{array}{c} D_{C}\left(A,B\right)=1-S_{C}\left(A,B\right), \end{array}$$

where *A* and *B* are two arbitrary vectors and *S* is the square of the distance between them.

However, this criterion is not an appropriate distance because it does not guarantee the principle of triangular inequality and goes beyond the principle of superposition. To compensate for the principle of triangular inequality, while maintaining the same order as before, it is necessary to convert it to an angular distance.

The cosine distance of two vectors can be converted into equation (4) with the Euclidean inner product formula:

(4) $$\begin{array}{c} A.B=\left\|A\right\|\left\|B\right\|\cos\;\theta , \end{array}$$

where *A* and *B* are two arbitrary vectors and *θ* is the angle between them.

And finally, by applying a series of changes and inner product, the criterion of cosine similarity used in this study can be expressed as

(5) $$\begin{array}{c} \text{similarity}=\cos\left(\theta \right)=\frac{\;A.\;B}{\left\|A\right\|\left\|B\right\|},\\ =\frac{\sum_{i=1}^{n}A_{i}\times B_{i}}{\sqrt{\sum_{i=1}^{n}{\left(A_{i}\right)}^{2}}\times \sqrt{\sum_{i=1}^{n}{\left(B_{i}\right)}^{2}}}, \end{array}$$

where *A* and *B* are two arbitrary vectors, *θ* are the angles between them, and *Ai* and *Bi* are components of the two vectors. The results of the above formula are between −1 and 1, in which −1 represents completely different, 1 represents completely similar, 0 indicates noncorrelation or orthogonality, and the values between them indicate the degree of similarity or dissimilarity.

After clustering, there is now a regular data set that is divided into 25 categories (clusters). Using the edge calculation algorithm, three larger values are calculated in each cluster, that is, 25 values of *A*, 25 values of *B*, and 25 values of *C.* The largest value of A in every 25 clusters is called the total A. Also, the largest value of B in a total of 25 clusters is called the value of total B, and the largest value of C in a total of 25 clusters is called the value of total C (Table 4).

As can be seen, total *A* (13.87) is created in cluster number 11, total *B* in cluster number 9, and total *C* in cluster number 10.

### 3.7. Degree of User Interaction in Each Cluster

**Figure 5:**
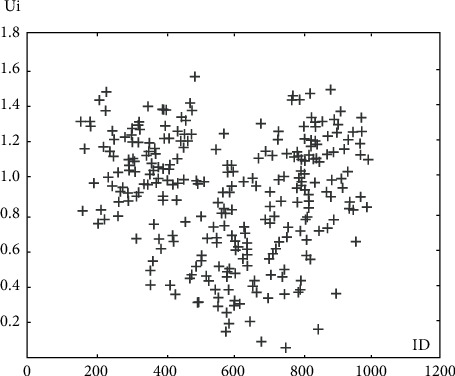
Degree of user interaction in the first cluster.

Here, the values of the UIi formula for nodes with different ID are shown. It can be said that whichever has the highest UIi value is selected as the most important node. The amount of user interactions in the first cluster is shown in Figure 5.

### 3.8. Score of Users in Each Cluster

**Figure 6:**
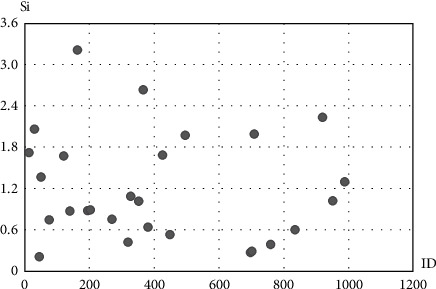
Score of users in the first cluster.

The score of users was obtained according to the community algorithm using the corresponding formula, for nodes with different IDs. In each cluster, each user with the highest score (Si) is considered the most important and effective nodes.  Figure 6 shows the score of users in the first cluster.

### 3.9. Impact of Users on the Network

**Figure 7:**
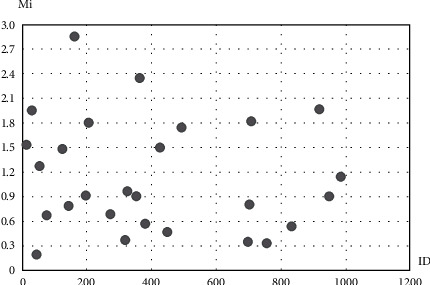
Degree of effectiveness of the first cluster's users.

The final results of the proposed method for identifying users who are effective in each community are presented in this section, which is a hybrid measure of two criteria of user interaction and the score of the user.  Figure 7 shows the users in the first cluster based on the amount of impact (Mi). Given how many users are considered to start diffusion, we can select the users who have the most impact (and are at the top of the chart) from each community and start diffusion through them.

### 3.10. Calculating the Accuracy of the Proposed Method and Comparing It with Existing Methods

The proposed method has an accuracy of 86.081, which is about 0.2% better than the basic research method with an accuracy of 66.081%. It should be noted that, in the proposed method, the community structure of the IoT is considered, the community is first selected, and then the proposed method was applied; this issue was not considered in the basic research.

**Figure 8:**
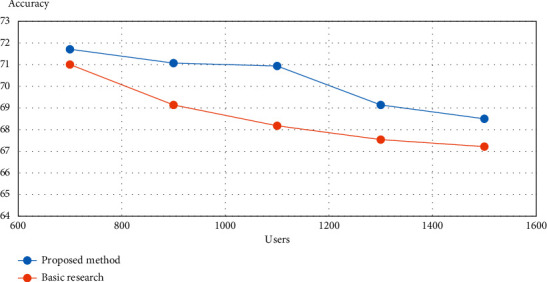
Comparing the accuracy of the proposed method and method [26].

The accuracy of the proposed method and the basic research for different numbers of users were compared, and as shown in Figure 8 and Table 5, the accuracy of the proposed method for different numbers has improved compared to the basic research.

It is observed that the accuracy of existing methods to find effective publishers decreases with the increasing number of users in IoT networks.

In order to start diffusion and diffusion information in basic methods, for example, 20 users are selected as initial acceptors to start diffusion information. All selected users may be in specific forums, and other forums may not share from publication; eventually the published messages may never reach them. However, in the proposed method, all forums are considered to start and at least one user is selected in each forum, and since this user is more influential and due to the similarity of his characteristics and desires with the users of the forum, this user can publish information and prevent the infiltration of information.

## 4. Conclusion

The IoMT is a connected infrastructure of medical devices, applications, and health systems and services. The rise of IoMT is driven by an increase in the number of connected medical devices that are able to generate, collect, analyze, or transmit health data or images and connect to healthcare provider networks, transmitting data to either a cloud repository or internal servers. This connectivity between medical devices means that security can no longer be examined within the neat, physical walls as it was considered before. Attacks on IoMT devices can potentially cause significant physical harm and life-threatening damage to the patients.

For addressing this problem, many researches have been done to provide a way for preventing IoMT intrusion and secure routing, in many of which IC and LT diffusion models start the diffusion process from nodes and have no assumptions about starting them. The publication process is not considered, and there is also a need to consider the possibility of accepting and receiving the publication which was done randomly. In other methods, it was based on publication or on popular topics, as they are limited in publication time. In later periods, they may lose their effectiveness and may not be popular in the future. So the methods used by popular users have become more popular for using in IoMT.

In degree-based methods, K-core and other ranking algorithms, including association algorithms, have no presuppositions about networks with association structure and cannot be deduced in networks with association structure. In the proposed method, users were placed in their respective forums, taking into account network forums that are based on individual characteristics in the user profile. Forums based on the characteristics of users' profiles, despite their advantages, are weak because users are reluctant to share their information with the public for security reasons.

## Figures and Tables

**Table 1:** An overview of previous models and the advantages and disadvantages of each.

Disadvantages	Advantages	Model (method)
Lack of privacy in some cases.	More accuracy and expansion of information in less time.	A model based on fuzzy logic, artificial intelligence, and gray wolf [9].

The process of searching and exchanging data is associated with problems in relatively large volumes of data.	More precision in the cluster social network.	Clustering model of social networks using multilayer perceptron network and gray wolf [10].

Dissemination of confidential information that owners are reluctant to disclose.	Wider use of social media, viral marketing, in which various forms of marketing message related to a company, brand, or product are transmitted with exponential growth, often through the use of social media applications.	A hybrid model of standard algorithm (naive k-means) and bee and gray wolf algorithm [11].

Time-consuming and repetitive results in the third stage and later, which is associated with high error.	Drawing a decision tree from data, expand the dissemination of information in social networking associations, break down a resource set into subcategories based on testing the value of an attribute.	Decision tree and gray wolf model [13].

There is a mapping of all related edges among the studied heads that the genetic algorithm is unable to examine.	Utilizing the capacity of social networks at many individual and social levels to identify problems and determine their solutions, establishing social relations, managing organizational affairs, policy-making, and guiding individuals on the path to achieving goals.	Model based on genetic algorithm and gray wolf [14].

Increased computational complexity compared to previous methods.	High accuracy and efficiency of the algorithm.	A model based on new genetic algorithm and gray wolf [15].

Occurrence of shilling attacks and loss of users' trust in recommender systems.	More speed in social networking forums.	Model based on participatory refinement algorithm and gray wolf [12].

Precise settings of input parameters that may be associated with errors.	Improving the quality of community discovery.	Model based on F algorithm and gray wolf [16].

Identifying edges statically, disregarding user interactions.	Maintaining data privacy.	Model based on a cuckoo optimization algorithm and gray wolf algorithm [17].

When the number of users is large, the number of errors in the algorithm exceeds the allowable limit defined in the default algorithm, and the results become invalid.	Improving the structure of users' social network, providing different services to users spatially.	Model based on a new algorithm based on spatial data and gray wolf [18].

Loss of time and place and more role of space in the social network and shaking the foundations of users' identities.	The social networks studied in the communication process can have positive effects.	Model based on genetic algorithm with a generational division and gray wolf [19].

The complexity of the implementation process and its time-consumption, the occurrence of human error in computing.	High accuracy and speed in social network processing.	A hybrid model of clustering and artificial neural network and gray wolf [20].

Lack of coverage of all nodes in the forum on large social networks.	Identifying the intermediate centrality of communication channels with anonymity and the characteristics of each anonymous node and effective communication channels, maintaining data privacy.	Combined model of anonymity clustering and genetic algorithm and gray wolf [21].

Inability to check all nodes and community members on social networks.	Identifying how information is disseminated on social networks, identifying key nodes in this type of network, providing quality control of information dissemination on social networks.	Model based on the evolutionary algorithm of bees and gray wolf [22].

Central network nodes cannot be checked by this method.	Identification and mapping of information exchange networks in social networking associations, revealing the main nodes.	Model based on modified genetic algorithm and gray wolf [23].

**Table 2:** A part of the study data set.

ID	Gender
1	1
2	0
3	0
4	0
5	1
6	1
7	1
8	1
9	0
10	1

**Table 3:** Factors related to set the parameters in MATLAB.

English name	Variable
Max Iteration	Number of iterations for clustering
Num Clusters	Number of clusters
Distance Function	Distance function

**Table 4:** Calculating the values of A, *B*, and *C* in each cluster.

C in each cluster	B in each cluster	A in each cluster	Cluster number
9.73	10.26	12.11	1
8.21	10.53	11.65	2
9.66	10.11	10.94	3
8.29	9.41	10.23	4
8.43	8.59	9.66	5
7.96	8.71	9.58	6
9.98	10.26	10.50	7
9.44	10.02	10.31	8
10.65	12.69	13.62	9
10.89	11.00	11.26	10
9.06	9.41	13.87	11
7.08	7.25	8.31	12
8.21	9.43	9.62	13
7.29	8.71	9.00	14
9.59	10.00	10.13	15
9.36	10.41	11.30	16
5.41	5.41	7.21	17
7.96	8.23	8.97	18
6.50	7.01	7.26	19
9.65	11.43	12.63	20
8.45	10.23	11.85	21
7.69	9.32	10.26	22
9.05	10.02	10.33	23
10.63	11.52	12.54	24
9.032	10.66	12.26	25
		= 13.87	Calculating total *A*, *B*, and C

**Table 5:** Details of comparison accuracy of the proposed method and method [26].

User number	Proposed method (%)	Basic research (%)
800	72	70.9
1000	71	68
1200	70.8	66.5
1400	68	65.5
1500	67	65

## Data Availability

Datasets of Mirai and Bashlite can be obtained from the following site: https://archive.ics.uci.edu/ml/datasets/detection_of_IoT_botnet_attacks_N_BaIoT.
